# Human monocyte-derived macrophages inhibit HCMV spread independent of classical antiviral cytokines

**DOI:** 10.1080/21505594.2018.1535785

**Published:** 2018-11-07

**Authors:** Jennifer Becker, Volker Kinast, Marius Döring, Christoph Lipps, Veronica Duran, Julia Spanier, Pia-Katharina Tegtmeyer, Dagmar Wirth, Luka Cicin-Sain, Antonio Alcamí, Ulrich Kalinke

**Affiliations:** a Institute for Experimental Infection Research, TWINCORE, Centre for Experimental and Clinical Infection Research, a joint venture between the Helmholtz Centre for Infection Research and the Hannover Medical School, Hannover, Germany; b Model Systems for Infection and Immunity, Helmholtz Centre for Infection Research, Braunschweig, Germany; c Department of Vaccinology, Helmholtz Centre for Infection Research, Braunschweig, Germany; d German Center for Infection Research (DZIF), Hannover-Braunschweig site, Germany; e Institute for Virology, Hannover Medical School, Hannover, Germany; f Centro de Biología Molecular Severo Ochoa, Consejo Superior de Investigaciones Científicas - Universidad Autónoma de Madrid, Madrid, Spain

**Keywords:** Human cytomegalovirus, macrophages, plasmacytoid dendritic cells, type I interferons, epithelial cells

## Abstract

Infection of healthy individuals with human cytomegalovirus (HCMV) is usually unnoticed and results in life-long latency, whereas HCMV reactivation as well as infection of newborns or immunocompromised patients can cause life-threatening disease. To better understand HCMV pathogenesis we studied mechanisms that restrict HCMV spread. We discovered that HCMV-infected cells can directly trigger plasmacytoid dendritic cells (pDC) to mount antiviral type I interferon (IFN-I) responses, even in the absence of cell-free virus. In contrast, monocyte-derived cells only expressed IFN-I when stimulated by cell-free HCMV, or upon encounter of HCMV-infected cells that already produced cell-free virus. Nevertheless, also in the absence of cell-free virus, i.e., upon co-culture of infected epithelial/endothelial cells and monocyte-derived macrophages (moMΦ) or dendritic cells (moDC), antiviral responses were induced that limited HCMV spread. The induction of this antiviral effect was dependent on cell-cell contact, whereas cell-free supernatants from co-culture experiments also inhibited virus spread, implying that soluble factors were critically needed. Interestingly, the antiviral effect was independent of IFN-γ, TNF-α, and IFN-I as indicated by cytokine inhibition experiments using neutralizing antibodies or the vaccinia virus-derived soluble IFN-I binding protein B18R, which traps human IFN-α and IFN-β. In conclusion, our results indicate that human macrophages and dendritic cells can limit HCMV spread by IFN-I dependent as well as independent mechanisms, whereas the latter ones might be particularly relevant for the restriction of HCMV transmission via cell-to-cell spread.

## Introduction

Currently, 60–100% of people in developed and developing countries are latently infected with human cytomegalovirus (HCMV) [1]. Nevertheless, most people are ill-informed about risks associated with HCMV infections. This is mainly due to the fact that in immunocompetent hosts HCMV infection is asymptomatic. However, HCMV reactivation or infection of immunocompromised individuals, such as organ transplant patients, HIV infected patients, or infants, can cause severe morbidity and mortality [1]. Importantly, HCMV is the leading cause for congenital infection related disabilities and abortions in newborns [2,3]. Furthermore, recent studies suggest that the constant immune stimulus provided by latent HCMV infection and reactivation may contribute to immunosenescence [4]. Thus, there is a clinical need to prevent HCMV infection; however, to date no effective HCMV vaccine is available. One major reason for this is that upon HCMV infection of the host a massive immune response is induced, which does not eliminate the virus, but instead drives the virus into latency. This is conferred by various HCMV encoded immune evasion mechanisms, which the virus developed during hundreds of thousands of years of coevolution with humanity [5,6]. This coevolution is also the reason why cytomegalovirus (CMV) shows high species-specificity, i.e., HCMV infects only humans and not rodents. Thus, HCMV infection experiments cannot be performed in mice, and instead murine cytomegalovirus (MCMV) is used to study *in vivo* pathogenesis of CMV. However, there are major differences between HCMV and MCMV, especially regarding their interactions with the immune system [5,7]. Thus, the knowledge about the pathogenicity of HCMV is still limited. Therefore, it is of particular relevance to study the interactions of HCMV with the human immune system.

Previous studies in the human and murine model revealed that type I interferons (IFN-I) play an essential role in the protection against CMV infection [8–11]. IFN-I not only induce an antiviral state upon triggering of the IFN-I receptor (IFNAR), which is expressed on every nucleated cell of the body, but they also activate and regulate adaptive immune responses [12,13]. Upon virus infection mainly myeloid cells, such as plasmacytoid dendritic cells (pDC) and classical dendritic cells (DC) or macrophages (MΦ), are known to produce IFN-I [14]. Previously we showed that HCMV stimulated pDC as well as monocyte-derived DC and MΦ mount strong IFN-I responses, which are induced by sensing of HCMV in a Toll-like receptor 9- or cyclic GMP/AMP synthase (cGAS)-dependent manner, respectively [15]. Interestingly, the magnitude of cGAS activation, as determined by intracellular concentrations of the second messenger cGAMP [16], correlated with the extent of HCMV infection of the respective cell subset [15]. This indicates that infection of monocyte-derived cells is a prerequisite to trigger cytosolic cGAS and thus to induce IFN-I responses. Myeloid cells are natural targets of HCMV infection [17,18]. However, they constitute only a minor fraction of the wide repertoire of different cell types that are infected by HCMV, including fibroblasts, muscle cells, hepatocytes, neurons, epithelial, and endothelial cells [18,19]. Moreover, myeloid cells presumably are not the first cell type that is infected upon HCMV entry into the host, as the virus has to cross epithelial/mucosal surfaces in order to enter the body. Mouse experiments showed that upon intravenous infection endothelial cells are initial targets of CMV, from where the virus further spreads into organs [20]. In cell culture HCMV has a long replication cycle of approximately 3 d [21,22]. Thus, during the first hours to days of HCMV infection myeloid cells might not be infected, although the virus is already present in the body. Therefore, it seems likely that innate immune cells developed means to detect and fight viruses that are present within infected cells. Indeed, there are several examples in the literature that pDC are stimulated by infected cells to mount IFN-I responses [23–25], and that such responses are sometimes even stronger than upon direct stimulation by cell-free virus [26]. Moreover, upon MCMV infection of mice an initial wave of IFN-I expression was detected already 4 h post infection that was followed by an even higher IFN-I wave after 36 h [27]. These results indicate that there are early detection and protection mechanisms in place. Furthermore, a murine *in vitro* study showed that bone marrow derived DC are able to efficiently reduce MCMV replication upon co-culture with infected endothelial cells or fibroblasts in an IFN-I dependent manner [28].

Here, we show that also human monocyte-derived macrophages and dendritic cells are able to successfully reduce HCMV spread when co-cultured with HCMV-infected epithelial or endothelial cells. Interestingly, under such conditions protection is conferred in an IFN-γ, TNF-α, and IFN-I independent manner.

## Results

### Upon co-culture with HCMV-infected cells pDC, but not monocyte-derived cells, mount abundant IFN-α responses

**Figure 1.**
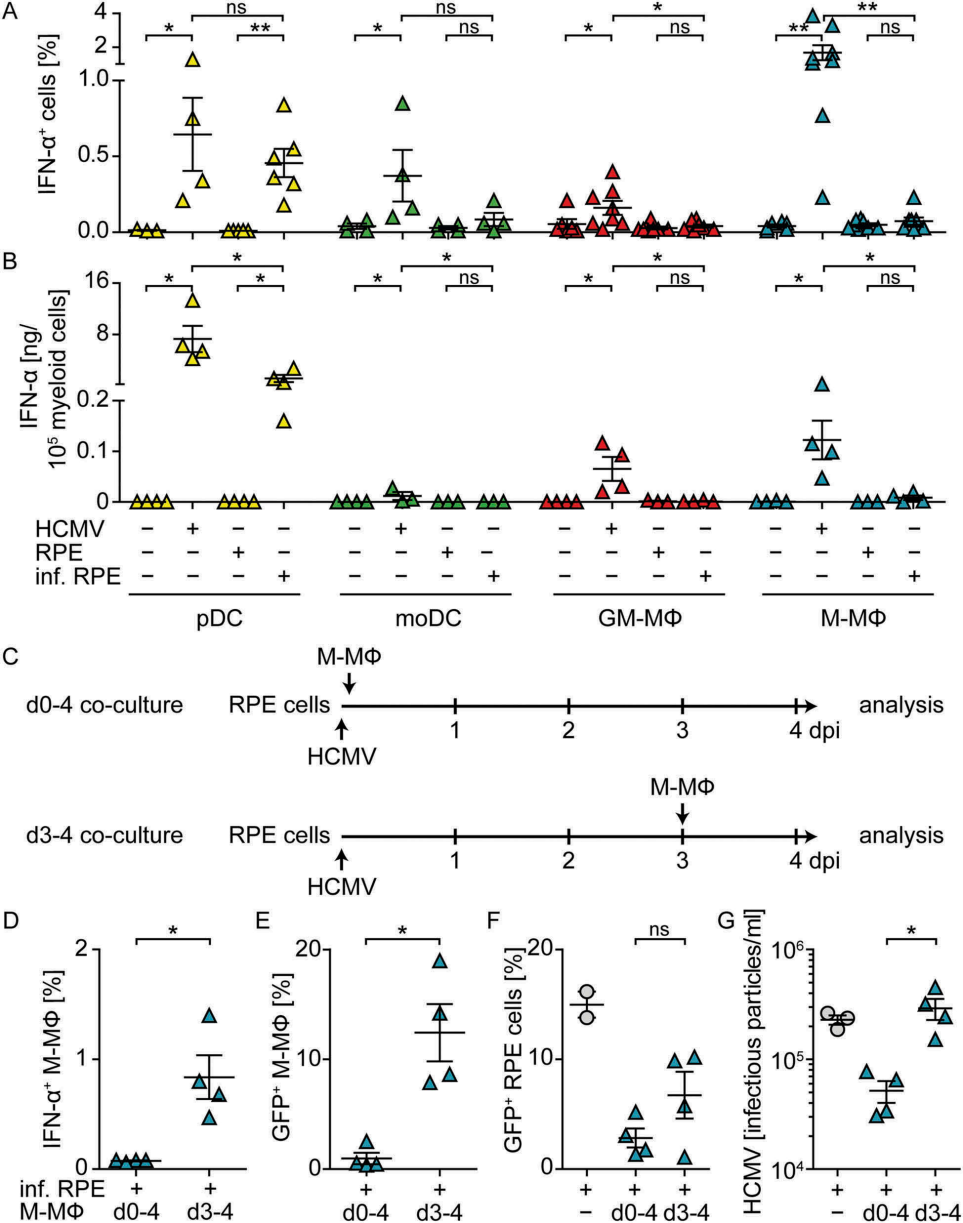
Co-culture of myeloid cells with HCMV-infected RPE cells induces enhanced IFN-α responses by pDC, but not by monocyte-derived cells. pDC, moDC, GM-CSF MΦ (GM-MΦ), or M-CSF MΦ (M-MΦ) were directly infected with HCMV-GFP at MOI 3. Furthermore, RPE cells were infected with HCMV at MOI 12, washed to remove cell-free virus, and co-cultured with myeloid cell subsets at a 1:4 ratio for 24 h and (a) percentages of intracellular IFN-α^+^ myeloid cells or (b) the IFN-α content in cell-free supernatants was determined by flow cytometry or an ELISA method, respectively. (c) RPE cells were infected with HCMV-GFP (MOI 12), washed, and co-cultured with M-CSF MΦ either from day (d) 0–4 post infection or from d 3–4 post infection and (d) percentages of intracellular IFN-α^+^ M-CSF MΦ, (e) HCMV-GFP^+^ M-CSF MΦ, (f) HCMV-GFP^+^ RPE cells, or (g) infectious HCMV particles in the supernatant of such cultures were determined. Mean ± SEM of (a) 4–8, (b) 3–4, or (d-g) 4 different donors from 2–4 independent experiments. inf. = infected, ns = not significant, *: p ≤ 0.05, **: p ≤ 0.01 (one-tailed Mann-Whitney test).

As reported previously by us and others [15,29–34], direct HCMV stimulation of pDC as well as monocyte-derived DC, GM-CSF MΦ, and M-CSF MΦ induced abundant IFN-α expression (Figure 1(a)) and secretion of IFN-α 24 hours post infection (hpi) (Figure 1(b)). To study whether also contact with HCMV-infected cells induced antiviral IFN-I responses, retinal pigment epithelial cells (RPE cells), which are permissive for HCMV replication [35,36], were used. RPE cells were infected with similar amounts of HCMV as used for direct infection of myeloid cells, washed to remove cell-free virus, and then co-cultured with the different myeloid cell subsets at a 1:4 ratio. Interestingly, under such conditions only pDC mounted high IFN-α responses, whereas the monocyte-derived cell subsets showed very little or no IFN-α expression 24 hpi (Figure 1(a,b)).

Presumably monocyte-derived cells are dependent on HCMV infection in order to activate cGAS and thus to mount IFN-α responses [15]. Therefore, we hypothesized that the lack of IFN-α responses by monocyte-derived cells stimulated with HCMV-infected cells might be due to the lack of cell-free virus particles that would infect myeloid cells. To test this, we prolonged the incubation time of co-cultures until 4 d post infection (dpi) and thus allowed the release of newly formed virus particles from HCMV-infected RPE cells, which is normally detected after 3 d of incubation [21,22]. We infected RPE cells, washed them, and directly incubated them with M-CSF MΦ for 4 d (d0-4 co-cultures, Figure 1(c)). Additionally, we infected RPE cells, washed them, incubated them for 3 d, and then added the M-CSF MΦ and incubated for another day (d3-4 co-cultures, Figure 1(c)). Interestingly, although cell-free virus was present in all cultures on 4 dpi (Figure 1(g)), in d0-4 co-cultures no IFN-α producing M-CSF MΦ (Figure 1(d)) and basically no HCMV-infected M-CSF MΦ (Figure 1(e)) were detected. Moreover, the percentage of HCMV-infected RPE cells (Figure 1(f)) as well as the number of released virus particles (Figure 1(g)) was lower in d0-4 co-cultures than in HCMV-infected RPE mono-cultures. In contrast, d3-4 co-cultures contained IFN-α producing (Figure 1(d)) and HCMV-infected M-CSF MΦ (Figure 1(e)). Also the percentage of HCMV-infected RPE cells (Figure 1(f)) as well as the release of infectious virus particles (Figure 1(g)) was substantially increased in d3-4 co-cultures when compared with d0-4 co-cultures. Taken together, these data showed that, in contrast to pDC, monocyte-derived cells did not mount abundant IFN-α responses when co-cultured with HCMV-infected RPE cells in the absence of cell-free virus. Interestingly, after 4 d of co-culturing M-CSF MΦ and HCMV-infected RPE cells, cell-free virus was produced; however at highly reduced titers compared with RPE mono-cultures. Nevertheless, if cell-free virus was present at the time of co-culture establishment, i.e., d3-4 co-cultures, monocyte-derived cells got infected and mounted IFN-α responses.

### Co-culture with monocyte-derived cells inhibits viral spread in HCMV-infected cells

**Figure 2.**
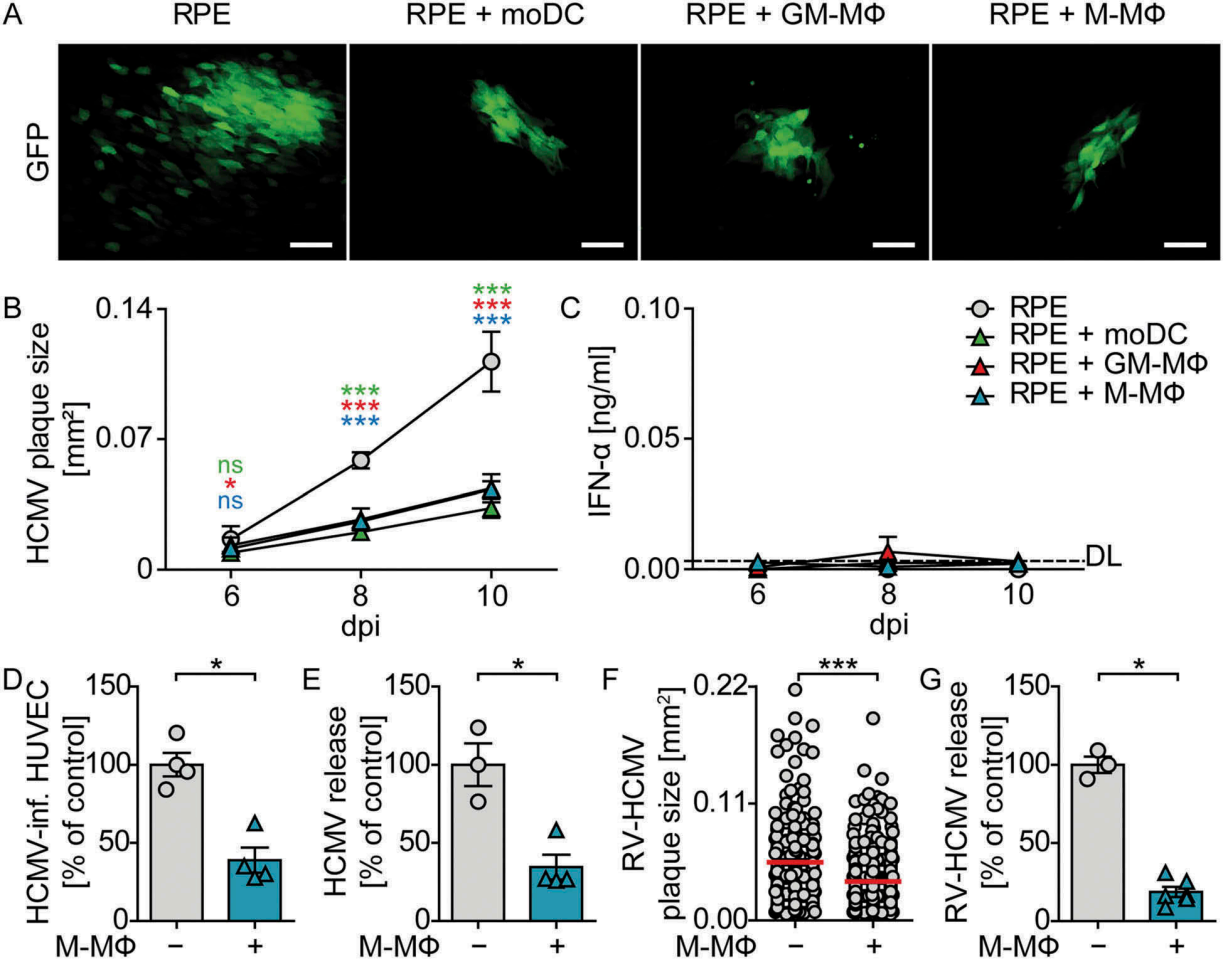
Co-culture of monocyte-derived cells with HCMV-infected RPE cells reduces viral spread. RPE cells were infected with HCMV-GFP at MOI 0.1, washed, and co-cultured with moDC, GM-CSF MΦ, or M-CSF MΦ at a 1:4 ratio. (a) 10 dpi HCMV plaque formation was analyzed by fluorescence microscopy (scale bar = 100 µm). (b) 6, 8, and 10 dpi the size of individual HCMV plaques was determined by using ImageJ software. (c) Secreted IFN-α was monitored by ELISA analysis of cell-free supernatants. HUVEC were infected with HCMV-GFP at MOI 1, co-cultured with M-CSF MΦ, and 10 dpi (d) HUVEC were analyzed for percentages of GFP expressing cells by flow cytometry and (e) supernatants were analyzed for the amount of infectious virus particles. RPE cells were infected with RV-TB40-BAC_KL7_-SE-EGFP (RV-HCMV), co-cultured with M-CSF MΦ, and 13 dpi (f) the size of individual HCMV plaques was determined and (g) supernatants were analyzed for the amount of infectious virus particles. Data in (d/e/g) show values as percent of control infections in HUVEC or RPE mono-cultures. Mean ±SEM of (b/c) 4–7 ((b) 26–102 plaques analyzed), (d/e) 4, and (f/g) 6 ((f) 212–368 plaques analyzed) different donors from 2–3 independent experiments. DL = detection limit, dpi = days post infection, *: p ≤ 0.05, ***: p ≤ 0.001 (one-tailed Mann-Whitney test).

To further test whether co-culture with monocyte-derived cells inhibited virus growth, we monitored plaque formation upon co-culture of moDC, GM-CSF MΦ, and M-CSF MΦ with HCMV-infected RPE cells during 10 d. RPE cells were infected with HCMV at MOI 0.1, washed to remove cell-free virus, and co-cultured with myeloid cells at a 1:4 ratio. As the virus expressed a GFP reporter under the control of the major immediate early promotor, GFP expression was analyzed by fluorescence microscopy as a measure of HCMV infection. 10 dpi HCMV-infected RPE cells showed HCMV plaques as well as several single HCMV-infected cells in close proximity of the plaques (Figure 2(a)). In contrast, upon co-culture of moDC, GM-CSF MΦ, or M-CSF MΦ with infected RPE cells the size of the HCMV plaques was significantly reduced and basically no single infected cells were detected (Figure 2(a)). Determination of the plaque size over time revealed that until 6 dpi the plaque size developed similarly in all cultures, whereas at later time points HCMV plaques were significantly smaller in co-cultures with myeloid cells than in RPE mono-cultures (Figure 2(b)). To study whether the reduced HCMV spread in the presence of myeloid cells was mediated by IFN-I, supernatants of co-cultures were analyzed for their IFN-α content. Surprisingly, IFN-α concentrations were only barely above the detection limit in all supernatants analyzed (Figure 2(c)), indicating that protection against HCMV was achieved independent of IFN-α. Moreover, we confirmed that the protection against HCMV was not limited to co-cultures of RPE and myeloid cells, as also HCMV infection of human umbilical vein endothelial cells (HUVEC) was significantly reduced upon co-culture with M-CSF MΦ (Figure 2(d,e)). Of note, the HCMV reporter virus that was used in the experiments above lacked several viral genes, including genes encoding the immune evasion molecules US2-US6, which were lost during the generation of a BAC clone of the virus strain TB40/E [22]. Importantly, also the repaired HCMV variant RV-HCMV, in which the lost viral genes were reconstituted [37], showed reduced spread in infected RPE cells upon co-culture with M-CSF MΦ (Figure 2(f)), although the effect was not as pronounced as in experiments with the HCMV variant lacking the immune evasion genes. Moreover, also RV-HCMV titers in the culture supernatant were more than 70% decreased in the presence of M-CSF MΦ (Figure 2(g)), indicating that the reconstituted immune evasion molecules did not inhibit the antiviral effect in co-cultures. Collectively, these data showed that co-culture of monocyte-derived cells with infected epithelial and endothelial cells inhibited HCMV spread independent of IFN-α.

### Protection against HCMV requires close contact between macrophages and infected epithelial cells

**Figure 3.**
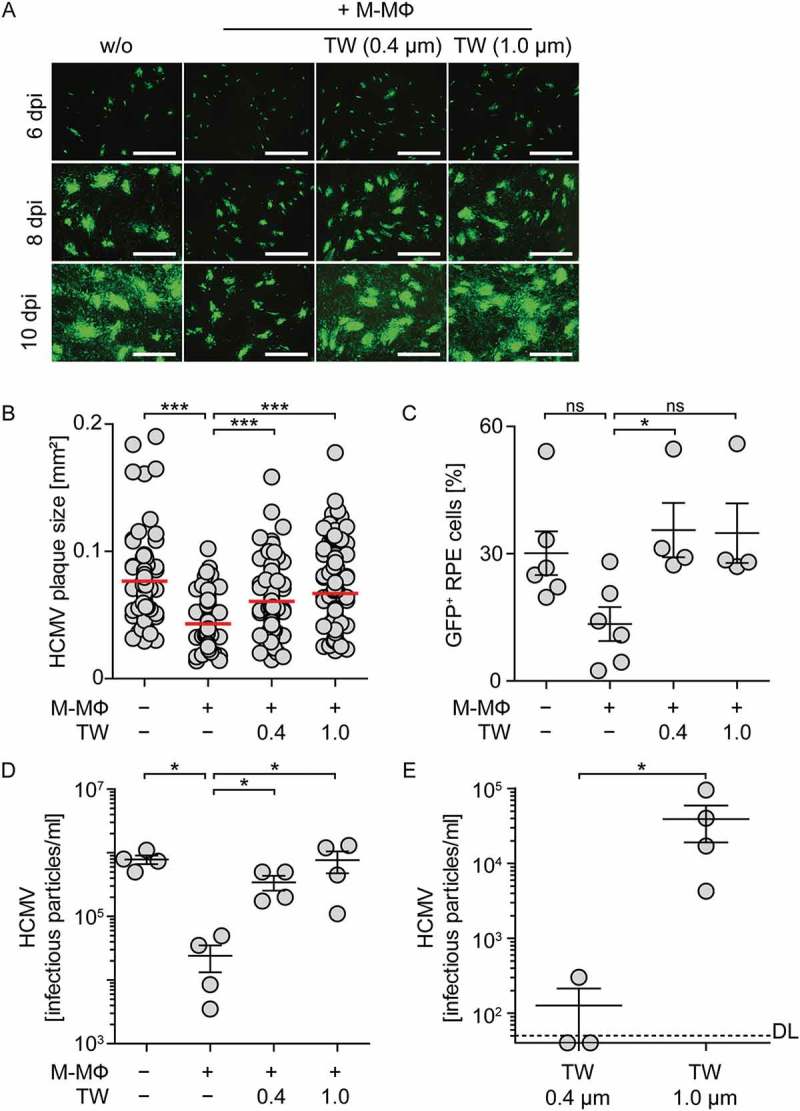
Inhibition of direct cell-cell contact between macrophages and infected epithelial cells impairs protection against HCMV. RPE cells were infected with HCMV-GFP at MOI 0.1, washed, and co-cultured with M-CSF MΦ at a 1:2 ratio. M-CSF MΦ were added directly onto RPE cells or into transwell inserts with pore sizes of 0.4 µm or 1.0 µm. (a) 6, 8, and 10 dpi HCMV plaque formation was analyzed by fluorescence microscopy (scale bar = 1 mm) and 10 dpi (b) the size of individual HCMV plaques or (c) percentage of HCMV-GFP^+^ RPE cells was determined. Numbers of infectious HCMV particles were determined 10 dpi in (d) co-cultures or in the RPE cell compartment of the transwell co-cultures as well as (e) in the transwell inserts, which contained the M-CSF MΦ. Mean size of (b) 77–119 plaques using 4 different donors or mean ±SEM of (c-e) 3–4 different donors from 2 independent experiments. DL = detection limit, ns = not significant, *: p ≤ 0.05, ***: p ≤ 0.001 (one-tailed Mann-Whitney).

We next aimed to understand whether direct cell-cell contact between myeloid cells and infected cells was needed to induce the antiviral effect. Therefore, M-CSF MΦ were physically separated from HCMV-infected RPE cells by using a transwell system with pore sizes of 0.4 µm or 1.0 µm to allow passage of soluble factors as well as of virus particles, but not of cells. Upon direct co-culture of HCMV-infected RPE cells and M-CSF MΦ at a 1:4 or 1:2 ratio, HCMV plaque size, percentage of HCMV-infected cells, and the release of cell-free virus was significantly reduced (Figure 3(a-d) and Sup. Figure 1(a-c)). In contrast, upon separation of M-CSF MΦ and RPE cells by using the transwell system the plaque size was significantly increased again (Figure 3(a,b)) and percentages of HCMV-infected cells were as high as in RPE mono-cultures (Figure 3(c)). Furthermore, also the amount of released virus in the lower well, which contained the RPE cells, was similar to HCMV-infected RPE mono-cultures (Figure 3(d)). Interestingly, analysis of HCMV particles in the transwell inserts revealed that HCMV was able to cross 1.0 µm pores readily, whereas in transwell inserts with a pore size of 0.4 µm basically no virus particles were detected (Figure 3(e)). Of note, when using a highly sensitive IFN-I reporter system, we found only barely measurable IFN-I activity in all cultures (Sup. Figure 1(d)). Thus, the observed antiviral effect against HCMV seemed to depend on direct cell-cell contact between M-CSF MΦ and infected RPE cells.

### Supernatants from co-cultures of macrophages and HCMV-infected epithelial cells show antiviral effects

**Figure 4.**
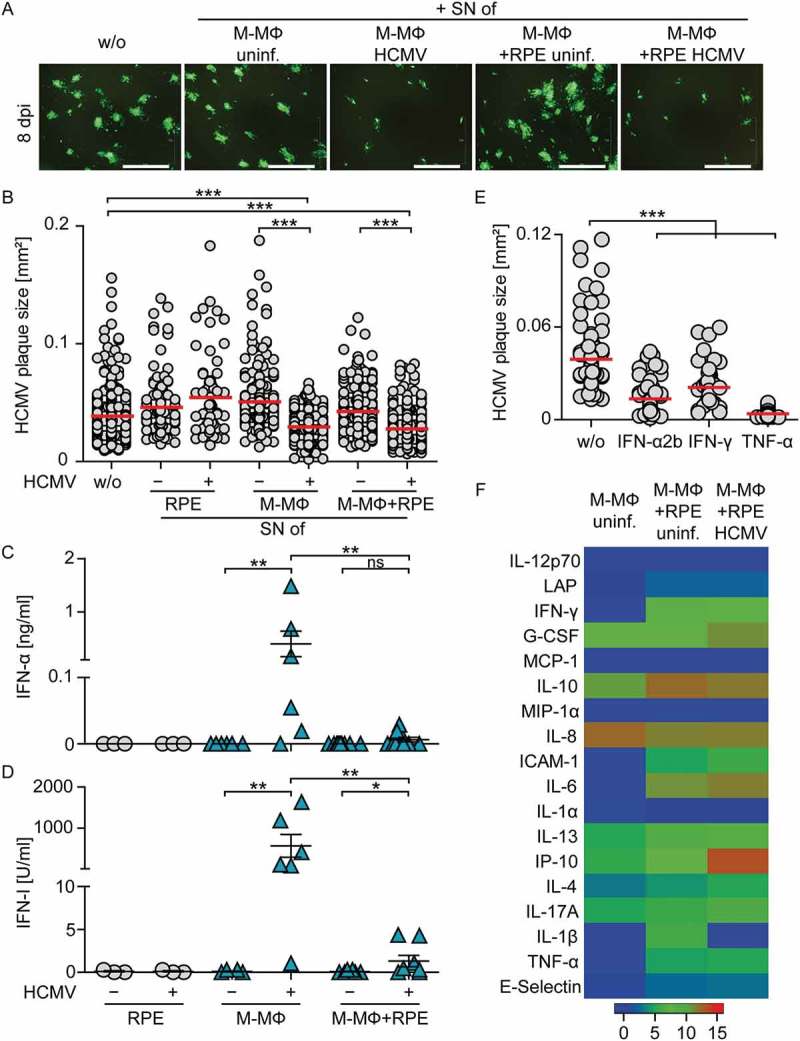
Supernatants from co-cultures of M-CSF MΦ and HCMV-infected RPE cells inhibit viral spread. Cell-free supernatants from uninfected or HCMV-infected RPE (MOI 12) or M-CSF-MΦ (MOI 3) mono-cultures and RPE/M-CSF-MΦ co-cultures were harvested 2 dpi and transferred onto fresh RPE cells infected with HCMV-GFP at MOI 0.1. (a) 8 dpi virus plaque formation was analyzed by fluorescence microscopy (scale bar = 1 mm) and (b) the size of individual plaques was determined. Cell-free supernatants prepared as described above were analyzed for (c) secreted IFN-α and (d) IFN-I activity using an ELISA method or an Mx2-Luc reporter cell line, respectively. (e) RPE cells were infected with HCMV-GFP at MOI 0.1 and incubated with recombinant (rec.) IFN-α2b, IFN-γ, or TNF-α for 8 d. Then the plaque size was analyzed. (f) Cell-free supernatants prepared as described above were analyzed for different secreted proteins using a bead-based cytokine array. Data visualize log (2) values of the measured cytokine concentrations in [pg/ml]. Mean size of (b) 67–371 plaques using 6 different donors or (e) 49–100 plaques from 3 independent experiments. Mean ±SEM of (c/d) 6 different donors from 3 independent experiments or data of (f) 3 different donors from 2 independent experiments. SN = supernatant, uninf. = uninfected, *: p ≤ 0.05, **: p ≤ 0.01, ***: p ≤ 0.001 (one-tailed Mann-Whitney test).

To understand whether the protection against HCMV was mediated by direct cell-cell contact alone, or whether secreted factors played a role, we tested the protective capacity of co-culture supernatants. Therefore, we transferred day 2 cell-free supernatant from co-cultures of M-CSF MΦ and HCMV-infected RPE cells on freshly infected RPE cells. Additionally, also supernatants from infected as well as uninfected mono-cultures and uninfected co-cultures were used. The quantification of plaque sizes after 8 d of incubation revealed that uninfected culture supernatants did not inhibit virus spread irrespective of whether they were derived from co- or mono-cultures (Figure 4(a,b)). As expected, supernatants from HCMV-infected M-CSF MΦ mono-cultures, which contained high levels of IFN-I (Figure 4(c,d)), decreased HCMV plaque size significantly (Figure 4(a,b)). Similarly, co-culture supernatants from M-CSF MΦ and HCMV-infected RPE cells significantly reduced virus spread (Figure 4(a,b)), although in these supernatants basically no IFN-α (Figure 4(c)) or IFN-I activity (Figure 4(d)) was detectable.

These results indicated that the protective activity in the supernatants from infected co-cultures was conferred by secreted factors different from IFN-I. To verify that also other cytokines such as IFN-γ and TNF-α can show antiviral activity [38–41], we treated HCMV-infected RPE cells with recombinant (rec.) IFN-α2b, IFN-γ, or TNF-α. Indeed, similar to treatment with rec. IFN-α2b, also treatment with rec. IFN-γ and rec. TNF-α significantly reduced the plaque size in HCMV-infected RPE cells after 8 d of incubation (Figure 4(e)). However, the analysis of culture supernatants revealed that although co-culture supernatants contained overall increased levels of secreted proteins compared with uninfected M-CSF MΦ mono-culture supernatants, neither IFN-γ nor TNF-α or other cytokines were dramatically upregulated in infected compared with non-infected co-culture supernatants (Figure 4(f)). Of the analyzed proteins, only IP-10 (CXCL-10) was massively enhanced in infected co-cultures (Figure 4(f)). Nevertheless, IP-10 was highly unlikely to promote the antiviral effect in supernatants from co-culture experiments as treatment with up to 100 ng/ml rec. IP-10 did not reduce HCMV plaque size (Sup. Figure 2). Collectively, these data indicate that the reduction of HCMV spread detected in co-cultures of macrophages and HCMV-infected cells was mediated by secreted factors, although an increase in the levels of classical antiviral cytokines was not detected.

### Protection against HCMV in co-cultures of macrophages and infected epithelial cells is not mediated by classical antiviral cytokines

**Figure 5.**
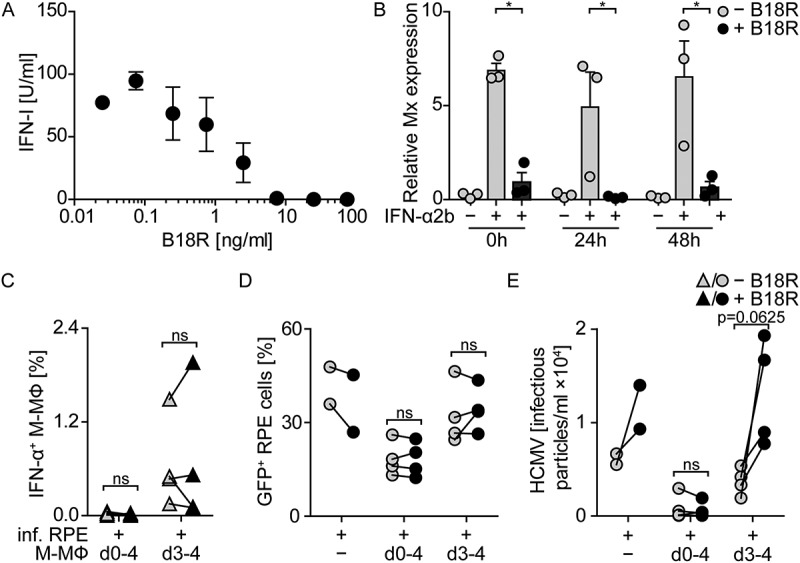
Co-cultures of macrophages and infected RPE cells incubated for four days show IFN-I independent inhibition of HCMV propagation. (a) Different concentrations of B18R were added to rec. IFN-α2b [100 U/ml] and after 1 h incubation the IFN-I activity was determined using an Mx2-Luc reporter cell line. (b) Co-cultures of M-CSF-MΦ and HCMV-infected RPE cells were set up, treated with 100 ng/ml B18R, and 0, 24, and 48 h later cultures were stimulated with 100 U/ml rec. IFN-α2b for 4 h and then Mx induction was analyzed by qPCR. (c-e) RPE cells were infected with HCMV-GFP at MOI 12, washed, and co-cultured with M-CSF MΦ from d 0–4 or d 3–4 post infection with (black symbols) or without (grey symbols) the daily addition of 100 ng/ml B18R. (c) Percentages of intracellular IFN-α^+^ M-CSF-MΦ, (d) HCMV-GFP^+^ RPE cells, or (e) infectious HCMV particles in the supernatants of such cultures were determined. Mean ±SEM of (a) 3 independent experiments and (b) 3 or (c/d/e) 4 different donors from 2 independent experiments. inf. = infected, *: p ≤ 0.05 (one-tailed Mann-Whitney test, (b)), ns = not significant (p ≥ 0.29) (one-tailed Wilcoxon signed rank test (c/d/e)).

Despite the fact that we found only barely measurable IFN-I amounts in the supernatants of infected co-cultures, our results also showed that treatment with rec. IFN-α2b efficiently inhibited HCMV spread (Figure 4(e)), even when used at very low concentrations such as 1–10 U/ml (data not shown). To exclude that low concentrations of IFN-I mediated protective effects in co-cultures of macrophages and infected RPE cells, we made use of the viral IFN-I binding protein from vaccinia virus, B18R. B18R attaches to the cell surface and catches human IFN-α as well as IFN-β with higher affinity than the endogenous receptor and thus efficiently inhibits IFNAR triggering by its cognate ligands [42–45]. Pretreatment with 7.5 ng/ml and higher concentrations of B18R inhibited the antiviral activity of 100 U/ml rec. IFN-α2b efficiently (Figure 5(a)). To test the stability of B18R in cell culture, RPE cells were infected with HCMV (MOI 0.1), macrophages were added, and the cells were treated with B18R (100 ng/ml). After incubation for 0, 24, and 48 h 100 U/ml rec. IFN-α2b was added and 4 h later induction of the interferon stimulated gene Mx was monitored. Under all conditions tested B18R efficiently inhibited Mx induction (Figure 5(b)) showing the stability of B18R in cell culture. Nevertheless, in the following co-culture experiments 100 ng/ml B18R was added daily to inevitably inhibit IFN-I.

We showed above that during 4 d co-cultivation of macrophages and HCMV-infected RPE cells (d0-4 co-cultures) the macrophages were not triggered to produce IFN-α and, nevertheless, release of infectious HCMV particles was decreased (see Figure 1(c-g)). In contrast, addition of macrophages to HCMV releasing RPE cells on day 3 post infection and subsequent co-cultivation for one day (d3-4 co-cultures) resulted in HCMV-infection and IFN-α production of the macrophages (see Figure 1(d,e)). To verify that in d0-4 co-cultures IFN-α did not contribute to the antiviral effect, we tested these conditions in the presence of B18R. Indeed, irrespective of whether B18R was added to d0-4 co-cultures or not, a similar reduction of the virus propagation was detected (Figure 5(d,e)). Furthermore, in d3-4 co-cultures addition of macrophages together with B18R did not impair IFN-α production of the macrophages (Figure 5(c)). Moreover, under such conditions B18R treatment increased HCMV particle formation, as similarly detected in B18R-treated RPE mono-cultures (Figure 5(e)). In conclusion, the inhibition of HCMV propagation in RPE cultures by M-CSF MΦ can be mediated by IFN-I when cell-free HCMV is present (d3-4 co-cultures), while it is independent of IFN-I when infected cells are encountered by M-CSF MΦ (d0-4 co-cultures).

**Figure 6.**
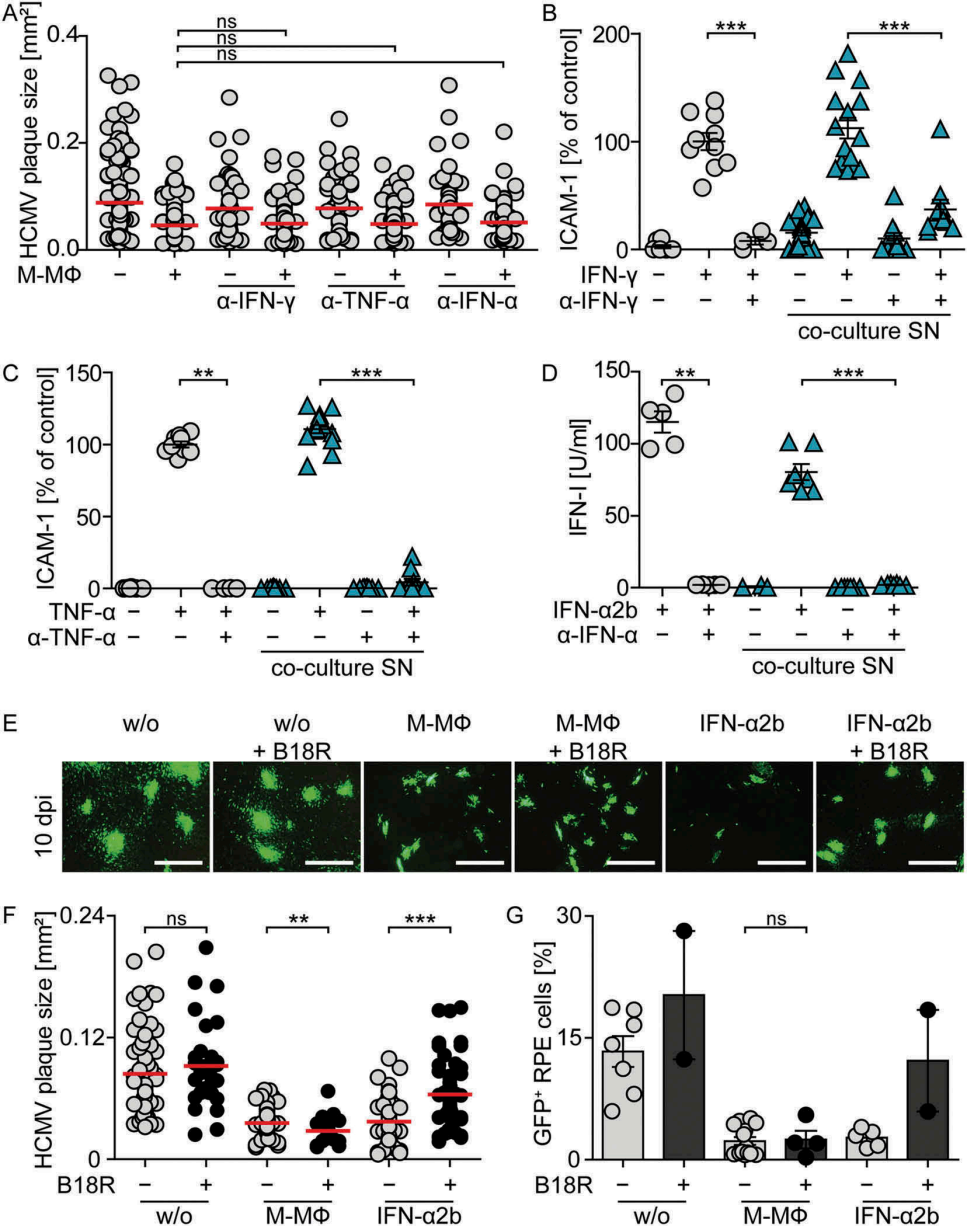
Inhibition of IFN-γ, TNF-α, or IFN-I does not impair protection against HCMV in co-cultures of macrophages and infected RPE cells. RPE cells were infected with HCMV-GFP at MOI 0.1, washed, and co-cultured with M-CSF MΦ with or without the addition of antibodies against human IFN-γ, TNF-α, or the IFN-I receptor. 10 dpi co-culture supernatants were harvested and (a) HCMV plaque formation was analyzed by fluorescence microscopy. To determine the remaining neutralizing potential of anti-human IFN-γ or TNF-α antibodies in day 10 co-culture supernatants, RPE cells were treated with (b) 100 U/ml rec. IFN-γ or (c) 10 ng/ml rec. TNF-α in the presence or absence of anti-human IFN-γ or TNF-α antibodies or the harvested day 10 co-culture supernatants, respectively, and upregulation of ICAM-1 expression was analyzed by flow cytometry. Data are shown as percent of ICAM-1 upregulation after IFN-γ or TNF-α treatment of RPE cells (control). (d) To determine the remaining potential of anti-human IFNAR antibodies to block IFN-I responses in day 10 co-culture supernatants, Mx2-Luc reporter cells were treated with 100 U/ml rec. IFN-α2b in the presence or absence of anti-human IFNAR antibodies or day 10 co-culture supernatants and IFN-I activity was measured. RPE cells were infected with HCMV-GFP at MOI 0.1, washed, and co-cultured with M-CSF MΦ with or without the addition of 100 ng/ml B18R daily. Furthermore, infected RPE cells were treated with 100 U/ml rec. IFN-α2b which was pretreated for 1 h with 100 ng/ml B18R. 10 dpi (e) HCMV plaque formation was analyzed by fluorescence microscopy (scale bar = 1 mm) and (f) the size of individual HCMV plaques or (g) percentages of HCMV-GFP^+^ RPE cells were determined. Mean size of (a) 116–184 or (f) 32–68 plaques using 2–7 different donors or mean ± SEM of (b/c) 7, (d) 5, and (g) 4 different donors from 2–3 independent experiments. ns = not significant, **: p ≤ 0.01, ***: p ≤ 0.001 (one-tailed Mann-Whitney test).

Finally, we addressed whether the observed plaque size reduction in co-culture experiments was impaired by IFN-I inhibition. To this end, we again used the B18R protein to inhibit IFN-I activity. Moreover, we deployed IFNAR-blocking antibodies as well as antibodies that neutralize IFN-γ or TNF-α, to also exclude involvement of minute levels of these classical antiviral cytokines. Indeed, neutralization of neither IFN-γ nor TNF-α influenced the reduction of plaque size in HCMV infected RPE cells co-cultured with M-CSF MΦ (Figure 6(a)), although the neutralizing effects of the antibodies were still evident even after 10 d of culture (Figure 6(b,c)). Similarly, although the activity of rec. IFN-α2b was inhibited by treatment with IFNAR-blocking antibodies (Figure 6(d)) or B18R (Figure 6(e,f)), such treatments did not affect the reduction of plaque size (Figure 6(a,e and f)) or the percentage of infected cells in co-cultures of M-CSF MΦ with infected RPE cells (Figure 6(g)). Thus, neither inhibition of IFN-γ or TNF-α nor efficient IFN-I inhibition did affect the protection against HCMV in co-cultures of macrophages and HCMV-infected epithelial cells.

## Discussion

To better understand HCMV pathogenicity it is of key relevance to gain an improved understanding of antiviral mechanisms that restrict HCMV dissemination. Previously we and others reported that CMV triggers human and murine immune cells to mount antiviral IFN-I responses, which are critical to protect against CMV infection [8–11,15,32–34]. Here we showed, that only pDC, but not monocyte-derived cells, were able to mount abundant IFN-I responses upon contact with HCMV-infected epithelial cells in the absence of cell-free virus. Nevertheless, monocyte-derived cells reduced HCMV spread, which was dependent on cell-cell contact between monocyte-derived cells and infected epithelial cells. We found that upon contact with infected cells antiviral factors were secreted into the supernatant, which inhibited the spread of HCMV. Importantly, this protection was independent of IFN-γ, TNF-α, or IFN-I. In conclusion, our data indicate that the immune system provides strategies to recognize infected cells and to subsequently mount IFN-I dependent as well as independent antiviral protection mechanisms.

pDC, which are known for their rapid and high IFN-I producing capacities [46–48], were able to recognize HCMV-infected epithelial cells and mount IFN-I responses even in the absence of cell-free virus particles. Similar findings have been reported for pDC in the context of other viral infections such as HCV or VSV in which case contact to virus-infected cells even increased IFN-I responses of pDC [23–25]. Thus, our results again highlight the huge capacity of pDC to recognize infections and rapidly mount IFN-I responses also in the context of infections with DNA viruses such as HCMV.

In contrast, monocyte-derived cells produced basically no IFN-I upon contact with infected epithelial cells, although abundant antiviral responses were induced that diminished HCMV infection in human epithelial and endothelial cells. These findings are in line with an earlier study in the murine system, in which co-culture of murine DC with MCMV-infected liver-sinusoidal endothelial cells or fibroblasts protected against MCMV infection [28]. Additionally, our experiments showed that upon physically separating human monocyte-derived macrophages (moMΦ) from infected epithelial cells by using transwell inserts with 0.4 or 1.0 µm pore sizes protection against HCMV was inhibited. Interestingly, also the presence of cell-free HCMV particles, which were able to pass through 1.0 µm pores, but basically not 0.4 µm pores, did neither influence protection nor induce IFN-I responses by the macrophages in the transwell inserts. Thus, the antiviral effect against HCMV seemed to be dependent on direct cell-cell contact between moMΦ and infected epithelial cells.

Our results further showed that protection could be conferred by co-culture supernatants of moMΦ and infected epithelial cells. Thus, we concluded that cell-cell contact induced the secretion of antiviral factors which then mediated protection against HCMV, although the involvement of some additional cell-cell contact dependent mechanisms could not be excluded. The analysis of several candidate proteins in the supernatant of uninfected and infected co-cultures revealed IP-10 to be highly enhanced, whereas most other cytokines were only slightly regulated. IP-10 has important chemokine functions in immunity such as the recruitment of antiviral T cells to the liver during MCMV infections [49,50]. Moreover, during infection of mice with the human herpes simplex virus 1 (HSV-1) IP-10 expression by epithelial cells was proposed as an early antiviral mechanism prior to IFN-I secretion, which contributes to the control of HSV-1 by recruitment of neutrophils [51]. Thus, our results are in accordance with the hypothesis that also in early HCMV infection cross-talk of myeloid cells with HCMV-infected cells induces abundant IP-10 responses, even in the absence of high HCMV titers. IP-10 expression may then contribute to the initiation of early antiviral immunity by recruitment of other immune cells. Nevertheless, IP-10 itself has not been reported to mediate direct antiviral effects and a reduction in HCMV spread upon treatment with rec. IP-10 was not observed in our system. While IP-10 is widely used as an indicator for IFN-γ or IFN-I activity as it is highly upregulated upon treatment with these cytokines [52,53], neither IFN-γ nor IFN-I were dramatically increased in the supernatant of infected co-cultures. However, even low level of classical antiviral cytokines such as IFN-γ, TNF-α, and particularly IFN-I can exhibit very potent effects. Indeed, results from the murine co-culture of DC with infected cells revealed IFN-I as a major mediator of protection against MCMV [28]. Moreover, in previous studies also human NK and T cells were shown to inhibit focal growth of HCMV in co-cultured cells [54–56]. NK cell derived IFN-γ was suggested to cause protection against HCMV [55]. Additionally, NK cells were suggested to induce IFN-β secretion of co-cultured, HCMV-infected fibroblasts, which subsequently mediated protection in concert with IFN-γ [57]. Nevertheless, we verified that in our system inhibition of HCMV propagation in epithelial cells by human moMΦ was mediated in an IFN-γ, TNF-α, and IFN-I independent manner. To show this, we made use of neutralizing antibodies against IFN-γ, TNF-α, or the IFN-I receptor, which did not affect inhibition of HCMV spread in epithelial cells by moMΦ. Moreover, we confirmed the independence on IFN-I receptor signaling by using a highly specific and potent soluble type I IFN binding protein of vaccinia virus, B18R. B18R was shown to potently inhibit human IFN-α and IFN-β responses [42–44]. In line with this, B18R treatment blocked the activity of up to 100 U/ml recombinant IFN-α2b, whereas the protective effect induced in HCMV-infected co-cultures was not affected. These data are in accordance with another recent study showing that human DC are able to inhibit HCMV infection in an IFN-I independent manner [58]. Collectively, our data showed that other factors than the classical antiviral cytokines IFN-γ, TNF-α, or IFN-I mediated protection against HCMV by moMΦ in the human system. Thus, there seem to be major differences in the antiviral mechanisms deployed by the various innate and adaptive immune cells of the human and murine system in combating HCMV or MCMV, respectively.

Previous studies which used clinical isolates of HCMV or an HCMV strain that was modified to mimic clinical isolates, suggested that clinically relevant HCMV preferably spreads on a cell-to-cell basis in the host and that, unlike cell-free virus infection, this cell-to-cell virus spread is resistant to IFN-I treatment [59,60]. In light of these findings, our results indicate that myeloid cells developed IFN-I independent antiviral restriction mechanisms to protect against HCMV transmission via direct cell-to-cell spread. Since we observed predominantly focal HCMV spread in RPE cells and the size of HCMV plaques was significantly reduced upon co-culture with monocyte-derived cells, the IFN-I independent protection we present here might be such a mechanism. Interestingly, this mechanism seemed to be only applied when moMΦ encountered infected cells in a state when cell-free virus particles were not present, yet. If the co-culture was established at such an early state, even upon prolonged co-culture and subsequent detection of cell-free virus in the supernatant, moMΦ were not infected and did not mount IFN-I responses, but continuously conferred IFN-I independent protection. This suggests that the soluble antiviral factor that reduced viral spread in the epithelial cells also reduced the vulnerability of macrophages to HCMV infection and thus impaired the activation of cGAS-mediated IFN-I responses by the macrophages. In contrast, when moMΦ were added to HCMV-infected cells that already produced cell-free virus, moMΦ were infected and produced abundant IFN-I responses, which are known to be effective against cell-free virus infection [59].

In conclusion, we showed that pDC are triggered by HCMV-infected cells to rapidly mount abundant IFN-I responses, even in the absence of cell-free virus particles. Importantly, also contact of monocyte-derived cells and infected cells induces abundant antiviral responses. However, these antiviral responses are independent of IFN-I and might be especially suited to restrict IFN-I-resistant transmission of HCMV via cell-to-cell spread. Additionally, by the induction of chemokines such as IP-10 other immune cells might be recruited to the site of infection. Nevertheless, upon progression of the infection and subsequent production of cell-free virus particles, monocyte-derived cells that are newly recruited might get infected and then are able to produce IFN-I which is effective against cell-free virus infection. Thus, our data contribute to an improved understanding of the diverse layers of protective mechanisms deployed by myeloid cells to combat HCMV infection.

## Materials and methods

### Cells and viruses

HCMV-GFP encoding a green fluorescent protein under the control of the major immediate early promoter was generated on the backbone of the endotheliotropic BAC-cloned TB40/E strain [22,61,62] and prepared as described previously [15]. The HCMV variant RV-TB40-BAC_KL7_-SE-EGFP is a repaired version of the TB40/E BAC virus, which contains an intact US region, a self-excisable BAC cassette, and an EGFP reporter under the control of the viral major immediate early promoter [37]. Human retinal pigment epithelial cells (RPE cells) and human fibroblasts (MRC-5 cells) were maintained in DMEM medium supplemented with 10% fetal calf serum. Human umbilical vein endothelial cells (HUVEC) were maintained in EGM-2 Bullet kit medium (Lonza, cc-3162).

### Determination of infectious virus particles

Confluent layers of MRC-5 cells were infected with culture supernatants and infection was enhanced by centrifugation at 300 *g* for 30 minutes. 3 dpi cells were fixed with ice-cold methanol and stained against cytomegalovirus immediate early and early nuclear proteins (Dako, M0854). Infected cells were visualized using a secondary goat anti-mouse-HRP antibody (KPL, 474–1806) and AEC substrate (Sigma, A6926-50TAB). HCMV-infected cells were counted and numbers of infectious particles were calculated.

### Primary cell isolation, differentiation, and stimulation

Plasmacytoid dendritic cells (pDC) as well as monocyte-derived cells were isolated and generated from blood samples of healthy donors as described previously [15]. In brief, pDC and CD14^+^ monocytes were isolated using ficoll density gradient centrifugation and magnetic activated cell sorting. CD14^+^ monocytes were differentiated for 5 d in serum-free DC medium (Cellgenix, 20801–0100) in the presence of 80 U/ml GM-CSF (granulocyte macrophage-colony stimulating factor, CellGenix, 1412–050), 100 ng/ml M-CSF (macrophage-colony stimulating factor, Miltenyi Biotec, 130–096-492), or 1000 U/ml GM-CSF and 1000 U/ml IL-4 (CellGenix, 1403–050) to obtain GM-CSF MФ, M-CSF MФ, or moDC, respectively.

### Infection of myeloid cells and co-culture of HCMV-infected cells and myeloid cells

pDC and monocyte-derived cells were infected with HCMV-GFP at MOI 3 (400.000 cells plus 1.2 × 10^6^ infectious particles of HCMV-GFP) and infection was enhanced by centrifugation at 300 *g* for 30 minutes. To study whether pDC and monocyte-derived cells were also activated by infected cells, we infected RPE cells at similar conditions as the myeloid cells (100.000 cells with 1.2 × 10^6^ infectious particles of HCMV-GFP, i.e., MOI 12), centrifuged at 300 *g* for 30 minutes, and 1.5 h later washed the cells. Then myeloid cells were added in CellGro DC medium (400.000 myeloid cells to 100.000 infected RPE cells, i.e., a ratio of 1:4). Cells and cell-free supernatants were harvested at the indicated time points for intracellular cytokine staining and ELISA or bead array analysis, as indicated.

Additionally, infection experiments were performed in which the spread of HCMV upon treatment of RPE cells with cytokines and co-culture supernatants or upon direct co-culture with monocyte-derived cells was evaluated. For the analysis of individual plaque sizes after several days of culture confluent RPE layers were infected with HCMV-GFP at MOI 0.1, centrifuged, and washed as described above. Subsequently, RPE cells were either treated with co-culture supernatants that were harvested from the co-culture experiments as described above at a 1:2 dilution in medium, or with recombinant IFN-α2b (100 U/ml) (IntronA, MSD), IFN-γ (100 U/ml) (PreproTech, 300–02), TNF-α (10 ng/ml) (PreproTech, 300-01A), or IP-10 (biolegend, 573502). To avoid dilution of the cytokines or supernatants, the medium was not replaced during the incubation time and the HCMV plaque size was analyzed when first fully formed plaques became detectable (8 dpi). Furthermore, RPE cells were infected with HCMV-GFP or RV-TB40-BAC_KL7_-SE-EGFP and co-cultured in CellGro DC medium with monocyte-derived cells at a 1:4 ratio. In these cultures the medium was exchanged regularly in order to allow the analysis of HCMV plaque sizes also at later time points, i.e., 10 or 13 dpi. Furthermore, cultures were set up with or without using a transwell system, in which RPE cells and moMФ were co-cultured at a 1:2 ratio. The transwell system was used in a manner that the lower chamber was seeded with normal numbers of RPE cells, i.e., 100.000 cells, whereas in the smaller insert there were seeded 200.000 myeloid cells. For co-culture experiments with HUVEC and moMФ, HUVEC were infected with HCMV-GFP at MOI 1 and co-cultured with moMФ in DMEM medium supplemented with 10% FCS.

### Cytokine analysis

Cell-free supernatants were analyzed by using Human IFN-alpha Platinum ELISA (eBioscience, BMS216TEN) or Human Inflammation 20plex FlowCytomix Multiplex Kit (eBioscience, BMS819FFRTU) according to the manufacturer’s instructions. To measure IFN-I activity Hela-Mx2Luc cells, which contain a BAC that encodes luciferase that is expressed under the control of the murine Mx2 promotor were used. Such cells are suited to detect human IFN-I as well as type III interferon activity. 4 × 10^4^ Hela-Mx2Luc cells were seeded in 96-wells and after 24 h of incubation the cells were stimulated with cell-free culture supernatants or rec. cytokines. As a reference for IFN-I activity rec. IFN-α2b was used at different concentrations ranging from 0.3 U/ml to 300 U/ml. 24 h later the cells were lysed in Glo Lysis Buffer (Promega, E2661) and luciferase expression was determined upon addition of luciferin (Promega, E263B) in the plate reader Synergy TM2 (Biotek).

### Flow cytometry analysis

Intracellular IFN-α staining was performed according to the intracellular staining protocol from BD Bioscience. Cells were treated with Brefeldin A (GolgiPlug, BD Bioscience, 555029) 6 h prior to harvesting and stained with an anti-human IFN-α antibody (Miltenyi Biotec, 130–092-602). RPE cells and monocyte-derived cells were distinguished by the expression of CD206 (anti-human CD206, biolegend, 321124) or CD163 (anti-human CD163, BD Bioscience, 556018) on monocyte-derived cells as well as CellTracker Violet BMQC (Invitrogen, C10094) staining of monocyte-derived cells prior to co-culturing. Data were acquired on a LSRII flow cytometer (BD Biosciences) and analyzed with FlowJo software (Tree Star).

### Inhibition of antiviral cytokine responses by neutralizing antibodies

In co-culture experiments of HCMV-infected RPE cells and monocyte-derived cells (for details see above) 10 µg/ml mouse anti-human IFN-γ (R&D Systems, MAB2851), 10 µg/ml goat anti-human TNF-α (R&D Systems, AF-210-NA), or 20 µg/ml mouse anti-human IFNAR Chain 2 (PBL, 21385–1) were added. Every 2–3 d one fourth of the medium was exchanged by fresh medium containing the respective types and concentrations of antibodies. After 10 d of incubation the co-culture supernatants were tested for their continued neutralizing potential of the added antibodies. In brief, 50 µl medium supplemented with 10 µg/ml of anti-human IFN-γ or TNF-α antibodies or 50 µl of co-culture supernatant were incubated for 1 h in a total volume of 100 µl containing 100 U/ml rec. IFN-γ or 10 ng/ml rec. TNF-α, respectively. Subsequently, mixtures were transferred to RPE cells and ICAM-1 upregulation was analyzed 24 h later by flow cytometry as a measure for IFN-γ and TNF-α activity. Similarly, 50 µl medium supplemented with 20 µg/ml anti-human IFNAR antibodies or 50 µl of co-culture supernatant were pre-incubated for 1 h with Hela-Mx2Luc cells. Subsequently, Hela-Mx2Luc cells were stimulated with 100 U/ml rec. IFN-α2b in a total volume of 100 µl for 24 h and IFN-I activity was determined as described above.

### Inhibition of IFN-I responses by B18R

Recombinant vaccinia virus-derived soluble IFN-I binding protein B18R, with a C-terminal V5-His tag, was produced in a baculovirus system and purified by affinity chromatography using NiNTA columns (Quiagen, 31014) as described previously [45]. 100 U/ml rec. IFN-α2b was pre-incubated for 1 h with increasing concentrations of B18R before measuring IFN-I activity by using Hela-Mx2-Luc cells. To evaluate B18R activity, co-cultures were established as described above and treated with a single dose of 100 ng/ml B18R. 100 U/ml rec. IFN-α2b was added either directly or after 24 h or 48 h and the mixtures were subsequently incubated for 4 h. Then, IFN-I activity was determined by analyzing endogenous Mx induction in co-cultures by qPCR. Furthermore, 100 U/ml rec. IFN-α2b either untreated or pretreated for 1 h with 100 ng/ml B18R was used to treat RPE cells that were infected with HCMV-GFP at MOI 0.1 and subsequently incubated for 10 d while one fourth of the medium was replaced with fresh medium daily. Co-cultures of HCMV-infected RPE cells and monocyte-derived cells were established as described above and 100 ng/ml B18R was added daily by replacement of one tenth (4-day co-culture) or one fourth (10-day co-culture) of medium with fresh medium containing B18R.

### qPCR

RNA extraction (Macherey-Nagel, 740955.250) and cDNA synthesis (Takara, RR036A) were performed according to the manufacturer’s instructions. 5 ng of cDNA was analyzed by quantitative PCR using Sensi-FAST SYBR no-ROX Kit (Bioline, BIO-98020) in a LightCycler 480 (Roche). All data are presented as expression relative to hypoxanthine phosphoribosyl transferase 1 (HPRT1) mRNA. The corresponding primers were:

HPRT1 forward, 5’-GAACGTCTTGCTCGAGATGTG-3’

HPRT1 reverse, 5’-CCAGCAGGTCAGCAAAGAATT-3’

Mx forward, 5’-ACAGGACCATCGGAATCTTG-3’

Mx reverse, 5’-CCCTTCTTCAGGTGGAACAC-3’

### Fluorescence microscopy

HCMV-GFP spread in RPE cells was monitored by fluorescence microscopy using the epi-fluorescence microscope IX-81 (Olympus) or the Eclipse TS100 (Nikon). The size of HCMV-GFP plaques was analyzed from pictures using ImageJ (v1.47) software.

### Statistical analysis

Data were statistically analyzed using the software package GraphPad Prism Version 5.0. In case of comparisons between multiple samples adjustments for multiple-comparisons were performed according to the Bonferroni method. Heat map analysis was generated using R software version 3.4.0.

## Abbreviations

HCMV: human cytomegalovirus; pDC: plasmacytoid dendritic cells; IFN: interferon; moMΦ: monocyte-derived macrophages; moDC: monocyte-derived dendritic cells; MCMV: murine cytomegalovirus; IFNAR: type I interferon receptor; cGAS: cyclic GMP/AMP synthase; cGAMP: cyclic GMP/AMP; GM-CSF: granulocyte macrophage-colony stimulating factor; M-CSF: macrophage-colony stimulating factor; RPE cells: retinal pigment epithelial cells; HSV-1: human herpes simplex virus 1; HUVEC: human umbilical vein endothelial cells; GFP: green fluorescent protein

## References

[1] Griffiths P, Baraniak I, Reeves M. The pathogenesis of human cytomegalovirus. J Pathol. 2015;235:288–297. 25205255 10.1002/path.4437

[2] Revello MG, Gerna G Diagnosis and management of human cytomegalovirus infection in the mother, fetus, and newborn infant. Clin Microbiol Rev. 2002;15:680–715. 12364375 10.1128/CMR.15.4.680-715.2002 PMC126858

[3] Sinclair J, Sissons P Latency and reactivation of human cytomegalovirus. J Gen Virol. 2006;87:1763–1779. 16760381 10.1099/vir.0.81891-0

[4] Pawelec G Immunosenenescence: role of cytomegalovirus. Exp Gerontol. 2014;54:1–5. 24291068 10.1016/j.exger.2013.11.010

[5] Loewendorf A, Benedict CA Modulation of host innate and adaptive immune defenses by cytomegalovirus: timing is everything. J Intern Med. 2010;267:483–501. 20433576 10.1111/j.1365-2796.2010.02220.x PMC2902254

[6] Hengel H, Brune W, Koszinowski UH Immune evasion by cytomegalovirus–survival strategies of a highly adapted opportunist. Trends Microbiol. 1998;6:190–197. 9614343 10.1016/s0966-842x(98)01255-4

[7] Rawlinson WD, Farrell HE, Barrell BG Analysis of the complete DNA sequence of murine cytomegalovirus. J Virol. 1996;70:8833–8849. 8971012 10.1128/jvi.70.12.8833-8849.1996 PMC190980

[8] Emodi G, O’Reilly R, Muller A, et al Effect of human exogenous leukocyte interferon in cytomegalovirus infections. J Infect Dis. 1976;133(Suppl):A199–204. 180200 10.1093/infdis/133.supplement_2.a199

[9] Wen Y, Liu P Interferon successfully inhibited refractory cytomegalovirus infection and resulted in CD4+ T-cells increase in a patient with AIDS. HIV Clin Trials. 2011;12:118–120. 21498155 10.1310/hct1202-118

[10] Presti RM, Pollock JL, Dal Canto AJ, et al Interferon gamma regulates acute and latent murine cytomegalovirus infection and chronic disease of the great vessels. J Exp Med. 1998;188:577–588. 9687534 10.1084/jem.188.3.577 PMC2212470

[11] Gil MP, Bohn E, O’Guin AK, et al Biologic consequences of Stat1-independent IFN signaling. Proc Natl Acad Sci U S A. 2001;98:6680–6685. 11390995 10.1073/pnas.111163898 PMC34412

[12] McNab F, Mayer-Barber K, Sher A, et al Type I interferons in infectious disease. Nat Rev Immunol. 2015;15:87–103. 25614319 10.1038/nri3787 PMC7162685

[13] Crouse J, Kalinke U, Oxenius A Regulation of antiviral T cell responses by type I interferons. Nat Rev Immunol. 2015;15:231–242. 25790790 10.1038/nri3806

[14] Swiecki M, Colonna M Type I interferons: diversity of sources, production pathways and effects on immune responses. Curr Opin Virol. 2011;1:463–475. 22440910 10.1016/j.coviro.2011.10.026 PMC3572907

[15] Paijo J, Doring M, Spanier J, et al cGAS senses human cytomegalovirus and induces type I interferon responses in human monocyte-derived cells. PLoS Pathog. 2016;12:e1005546. 27058035 10.1371/journal.ppat.1005546 PMC4825940

[16] Paijo J, Kaever V, Kalinke U cGAMP quantification in virus-infected human monocyte-derived cells by HPLC-coupled tandem mass spectrometry. Methods Mol Biol. 2017;1656:153–166. 28808968 10.1007/978-1-4939-7237-1_9

[17] Sinzger C, Plachter B, Grefte A, et al Tissue macrophages are infected by human cytomegalovirus in vivo. J Infect Dis. 1996;173:240–245. 8537667 10.1093/infdis/173.1.240

[18] Sinzger C, Digel M, Jahn G Cytomegalovirus cell tropism. Curr Top Microbiol Immunol. 2008;325:63–83. 18637500 10.1007/978-3-540-77349-8_4

[19] Sinzger C, Grefte A, Plachter B, et al Fibroblasts, epithelial cells, endothelial cells and smooth muscle cells are major targets of human cytomegalovirus infection in lung and gastrointestinal tissues. J Gen Virol. 1995;76(Pt 4):741–750. 9049319 10.1099/0022-1317-76-4-741

[20] Sacher T, Mohr CA, Weyn A, et al The role of cell types in cytomegalovirus infection in vivo. Eur J Cell Biol. 2012;91:70–77. 21492952 10.1016/j.ejcb.2011.02.002

[21] Sampaio KL, Cavignac Y, Stierhof YD, et al Human cytomegalovirus labeled with green fluorescent protein for live analysis of intracellular particle movements. J Virol. 2005;79:2754–2767. 15708994 10.1128/JVI.79.5.2754-2767.2005 PMC548422

[22] Sinzger C, Hahn G, Digel M, et al Cloning and sequencing of a highly productive, endotheliotropic virus strain derived from human cytomegalovirus TB40/E. J Gen Virol. 2008;89:359–368. 18198366 10.1099/vir.0.83286-0

[23] Dreux M, Garaigorta U, Boyd B, et al Short-range exosomal transfer of viral RNA from infected cells to plasmacytoid dendritic cells triggers innate immunity. Cell Host Microbe. 2012;12:558–570. 23084922 10.1016/j.chom.2012.08.010 PMC3479672

[24] Grabski E, Wappler I, Pfaender S, et al Efficient virus assembly, but not infectivity, determines the magnitude of hepatitis C virus-induced interferon alpha responses of plasmacytoid dendritic cells. J Virol. 2015;89:3200–3208. 25552725 10.1128/JVI.03229-14 PMC4337522

[25] Bruni D, Chazal M, Sinigaglia L, et al Viral entry route determines how human plasmacytoid dendritic cells produce type I interferons. Sci Signal. 2015;8:ra25. 25737587 10.1126/scisignal.aaa1552

[26] Frenz T, Graalmann L, Detje CN, et al Independent of plasmacytoid dendritic cell (pDC) infection, pDC triggered by virus-infected cells mount enhanced type I IFN responses of different composition as opposed to pDC stimulated with free virus. J Immunol. 2014;193:2496–2503. 25070849 10.4049/jimmunol.1400215

[27] Schneider K, Loewendorf A, De Trez C, et al Lymphotoxin-mediated crosstalk between B cells and splenic stroma promotes the initial type I interferon response to cytomegalovirus. Cell Host Microbe. 2008;3:67–76. 18312841 10.1016/j.chom.2007.12.008 PMC2703178

[28] Holzki JK, Dag F, Dekhtiarenko I, et al Type I interferon released by myeloid dendritic cells reversibly impairs cytomegalovirus replication by inhibiting immediate early gene expression. J Virol. 2015;89:9886–9895. 26202227 10.1128/JVI.01459-15 PMC4577895

[29] Doring M, Lessin I, Frenz T, et al M27 expressed by cytomegalovirus counteracts effective type I interferon induction of myeloid cells but not of plasmacytoid dendritic cells. J Virol. 2014;88:13638–13650. 25231302 10.1128/JVI.00216-14 PMC4248974

[30] Gredmark S, Tilburgs T, Soderberg-Naucler C Human cytomegalovirus inhibits cytokine-induced macrophage differentiation. J Virol. 2004;78:10378–10389. 15367604 10.1128/JVI.78.19.10378-10389.2004 PMC516431

[31] Romo N, Magri G, Muntasell A, et al Natural killer cell-mediated response to human cytomegalovirus-infected macrophages is modulated by their functional polarization. J Leukocyte Biol. 2011;90:717–726. 21742939 10.1189/jlb.0311171

[32] Kvale EO, Dalgaard J, Lund-Johansen F, et al CD11c+ dendritic cells and plasmacytoid DCs are activated by human cytomegalovirus and retain efficient T cell-stimulatory capability upon infection. Blood. 2006;107:2022–2029. 16269620 10.1182/blood-2005-05-2016

[33] Varani S, Cederarv M, Feld S, et al Human cytomegalovirus differentially controls B cell and T cell responses through effects on plasmacytoid dendritic cells. J Immunol. 2007;179:7767–7776. 18025223 10.4049/jimmunol.179.11.7767

[34] Chang WL, Barry PA, Szubin R, et al Human cytomegalovirus suppresses type I interferon secretion by plasmacytoid dendritic cells through its interleukin 10 homolog. Virology. 2009;390:330–337. 19524994 10.1016/j.virol.2009.05.013 PMC2747589

[35] Detrick B, Rhame J, Wang Y, et al Cytomegalovirus replication in human retinal pigment epithelial cells. Altered expression of viral early proteins. Invest Ophthalmol Vis Sci. 1996;37:814–825. 8603866

[36] Miceli MV, Newsome DA, Novak LC, et al Cytomegalovirus replication in cultured human retinal pigment epithelial cells. Curr Eye Res. 1989;8:835–839. 2551575 10.3109/02713688909000873

[37] Sampaio KL, Weyell A, Subramanian N, et al A TB40/E-derived human cytomegalovirus genome with an intact US-gene region and a self-excisable BAC cassette for immunological research. BioTechniques. 2017;63:205–214. 29185920 10.2144/000114606

[38] Schroder K, Hertzog PJ, Ravasi T, et al Interferon-gamma: an overview of signals, mechanisms and functions. J Leukoc Biol. 2004;75:163–189. 14525967 10.1189/jlb.0603252

[39] Davignon JL, Castanie P, Yorke JA, et al Anti-human cytomegalovirus activity of cytokines produced by CD4+ T-cell clones specifically activated by IE1 peptides in vitro. J Virol. 1996;70:2162–2169. 8642638 10.1128/jvi.70.4.2162-2169.1996 PMC190054

[40] Karupiah G, Xie QW, Buller RM, et al Inhibition of viral replication by interferon-gamma-induced nitric oxide synthase. Science. 1993;261:1445–1448. 7690156 10.1126/science.7690156

[41] Ruby J, Bluethmann H, Peschon JJ Antiviral activity of tumor necrosis factor (TNF) is mediated via p55 and p75 TNF receptors. J Exp Med. 1997;186:1591–1596. 9348317 10.1084/jem.186.9.1591 PMC2199110

[42] Colamonici OR, Domanski P, Sweitzer SM, et al Vaccinia virus B18R gene encodes a type I interferon-binding protein that blocks interferon alpha transmembrane signaling. J Biol Chem. 1995;270:15974–15978. 7608155 10.1074/jbc.270.27.15974

[43] Symons JA, Alcami A, Smith GL Vaccinia virus encodes a soluble type I interferon receptor of novel structure and broad species specificity. Cell. 1995;81:551–560. 7758109 10.1016/0092-8674(95)90076-4

[44] Alcami A, Symons JA, Smith GL The vaccinia virus soluble alpha/beta interferon (IFN) receptor binds to the cell surface and protects cells from the antiviral effects of IFN. J Virol. 2000;74:11230–11239. 11070021 10.1128/jvi.74.23.11230-11239.2000 PMC113220

[45] Montanuy I, Alejo A, Alcami A Glycosaminoglycans mediate retention of the poxvirus type I interferon binding protein at the cell surface to locally block interferon antiviral responses. FASEB J. 2011;25:1960–1971. 21372110 10.1096/fj.10-177188 PMC3101028

[46] Ito T, Kanzler H, Duramad O, et al Specialization, kinetics, and repertoire of type 1 interferon responses by human plasmacytoid predendritic cells. Blood. 2006;107:2423–2431. 16293610 10.1182/blood-2005-07-2709

[47] Barchet W, Cella M, Odermatt B, et al Virus-induced interferon alpha production by a dendritic cell subset in the absence of feedback signaling in vivo. J Exp Med. 2002;195:507–516. 11854363 10.1084/jem.20011666 PMC2193622

[48] Siegal FP, Kadowaki N, Shodell M, et al The nature of the principal type 1 interferon-producing cells in human blood. Science. 1999;284:1835–1837. 10364556 10.1126/science.284.5421.1835

[49] Delale T, Paquin A, Asselin-Paturel C, et al MyD88-dependent and -independent murine cytomegalovirus sensing for IFN- release and initiation of immune responses in vivo. J Immunol. 2005;175:6723–6732. 16272328 10.4049/jimmunol.175.10.6723

[50] Hokeness KL, Deweerd ES, Munks MW, et al CXCR3-dependent recruitment of antigen-specific T lymphocytes to the liver during murine cytomegalovirus infection. J Virol. 2007;81:1241–1250. 17108043 10.1128/JVI.01937-06 PMC1797530

[51] Iversen MB, Reinert LS, Thomsen MK, et al An innate antiviral pathway acting before interferons at epithelial surfaces. Nat Immunol. 2016;17:150–158. 26595890 10.1038/ni.3319

[52] Buttmann M, Berberich-Siebelt F, Serfling E, et al Interferon-beta is a potent inducer of interferon regulatory factor-1/2-dependent IP-10/CXCL10 expression in primary human endothelial cells. J Vasc Res. 2007;44:51–60. 17167270 10.1159/000097977

[53] Liu M, Guo S, Hibbert JM, et al CXCL10/IP-10 in infectious diseases pathogenesis and potential therapeutic implications. Cytokine Growth Factor Rev. 2011;22:121–130. 21802343 10.1016/j.cytogfr.2011.06.001 PMC3203691

[54] Sinzger C, Mangin M, Weinstock C, et al Effect of serum and CTL on focal growth of human cytomegalovirus. J Clin Virol. 2007;38:112–119. 17204454 10.1016/j.jcv.2006.11.009

[55] Wu Z, Sinzger C, Reichel JJ, et al Natural killer cells can inhibit the transmission of human cytomegalovirus in cell culture by using mechanisms from innate and adaptive immune responses. J Virol. 2015;89:2906–2917. 25540381 10.1128/JVI.03489-14 PMC4325726

[56] Jackson SE, Mason GM, Okecha G, et al Diverse specificities, phenotypes, and antiviral activities of cytomegalovirus-specific CD8+ T cells. J Virol. 2014;88:10894–10908. 25008941 10.1128/JVI.01477-14 PMC4178885

[57] Iversen AC, Norris PS, Ware CF, et al Human NK cells inhibit cytomegalovirus replication through a noncytolytic mechanism involving lymphotoxin-dependent induction of IFN-beta. J Immunol. 2005;175:7568–7574. 16301666 10.4049/jimmunol.175.11.7568

[58] Kasmapour B, Kubsch T, Rand U, et al Myeloid dendritic cells repress human cytomegalovirus gene expression and spread by releasing interferon-unrelated soluble antiviral factors. J Virol. 2018;92(1). 10.1128/JVI.01138-17 PMC5730771 29046460

[59] Murrell I, Bedford C, Ladell K, et al The pentameric complex drives immunologically covert cell-cell transmission of wild-type human cytomegalovirus. Proc Natl Acad Sci U S A. 2017;114:6104–6109. 28533400 10.1073/pnas.1704809114 PMC5468625

[60] Sinzger C, Knapp J, Plachter B, et al Quantification of replication of clinical cytomegalovirus isolates in cultured endothelial cells and fibroblasts by a focus expansion assay. J Virol Methods. 1997;63:103–112. 9015280 10.1016/s0166-0934(97)02082-x

[61] Riegler S, Hebart H, Einsele H, et al Monocyte-derived dendritic cells are permissive to the complete replicative cycle of human cytomegalovirus. J Gen Virol. 2000;81:393–399. 10644837 10.1099/0022-1317-81-2-393

[62] Borst EM, Standker L, Wagner K, et al A peptide inhibitor of cytomegalovirus infection from human hemofiltrate. Antimicrob Agents Chemother. 2013;57:4751–4760. 23856778 10.1128/AAC.00854-13 PMC3811406

