# Supplementary material for: Human monocyte-derived macrophages inhibit HCMV spread independent of classical antiviral cytokines
Source: Virulence. 2018 Nov 7;9(1):1669–84. doi: 10.1080/21505594.2018.1535785 (PMC7000197; doi:10.1080/21505594.2018.1535785)
Supplement: Supplemental Material [file kvir-09-01-1535785-g000.docx]

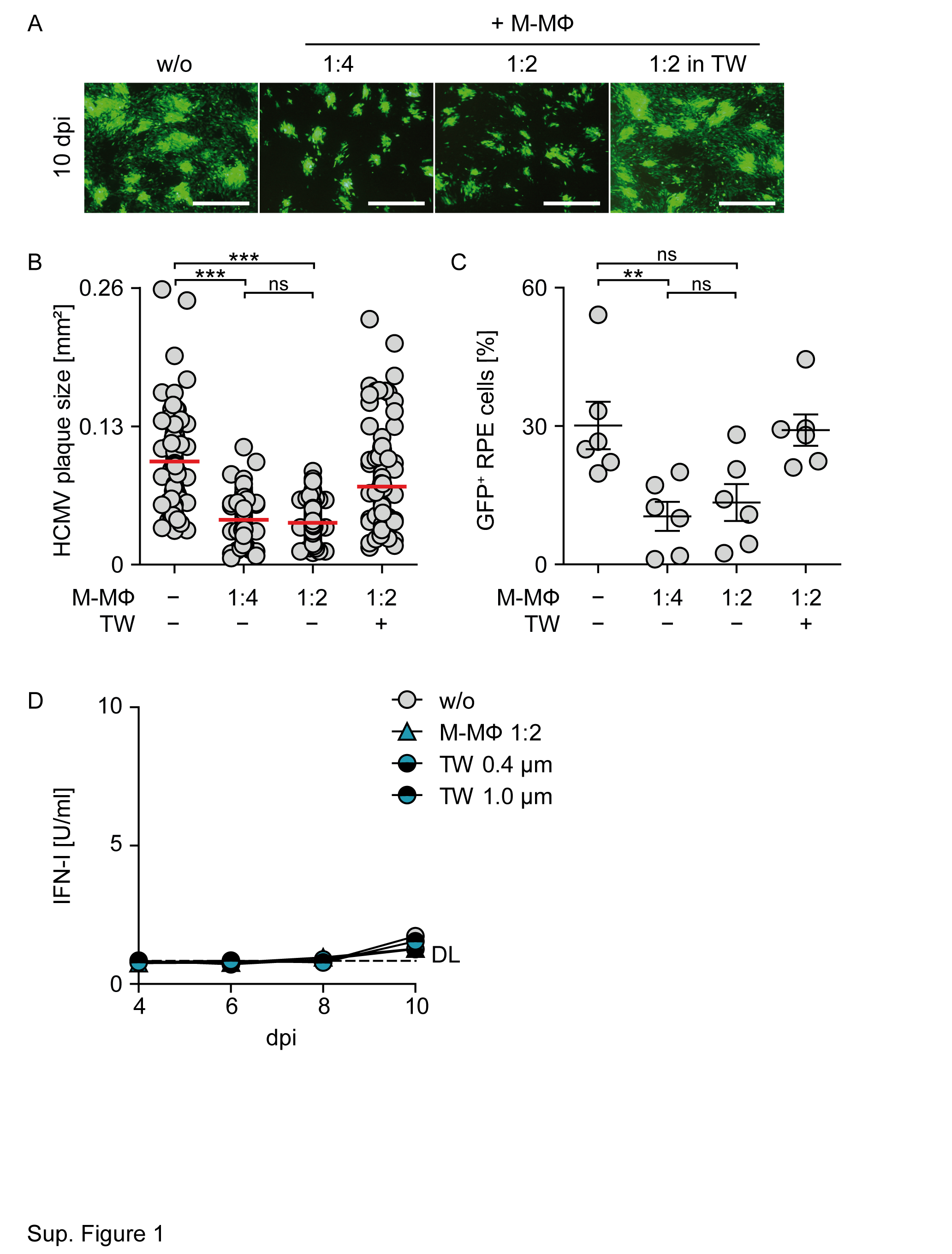


**Sup. Figure 1: Irrespective of whether HCMV-infected RPE cells and M-CSF MΦ were co-cultured at a ratio of 1:4 or 1:2, the virus spread was similarly impaired.**

RPE cells were infected with HCMV-GFP at MOI 0.1 and co-cultured with M-CSF MΦ at a 1:4 or 1:2 ratio. M-CSF MΦ were added either directly to RPE cells or into transwell inserts with a 0.4 µm pore size. (**A**) 10 dpi HCMV plaque formation was analyzed by fluorescence microscopy (scale bar = 1 mm) and (**B**) the size of individual plaques and (**C**) percentage of HCMV-GFP^+^ RPE cells was determined. (**D**) RPE cells were infected with HCMV-GFP at MOI 0.1 and co-cultured with M-CSF MΦ at a 1:2 ratio. M-CSF MΦ were added directly to RPE cells or into transwell inserts with pore sizes of 0.4 µm or 1.0 µm. Cell-free supernatants were analyzed for IFN-I activity using an Mx2-Luc reporter cell line. Mean size of (**B**) 34 – 39 plaques using 4 different donors or mean ± SEM of (**C/D**) 4 different donors from 2 independent experiments. DL = detection limit as defined by background level of unstimulated Mx2-luc reporter cells, ns = not significant, *: p ≤ 0.05, **: p ≤ 0.01, ***: p ≤ 0.001 (one-tailed Mann-Whitney), data of cultures without M-CSF MΦ and of co-cultures with M-CSF MΦ at a 1:2 ratio in (**B**/**C**) depict the same data as in Figure 3 B/C.


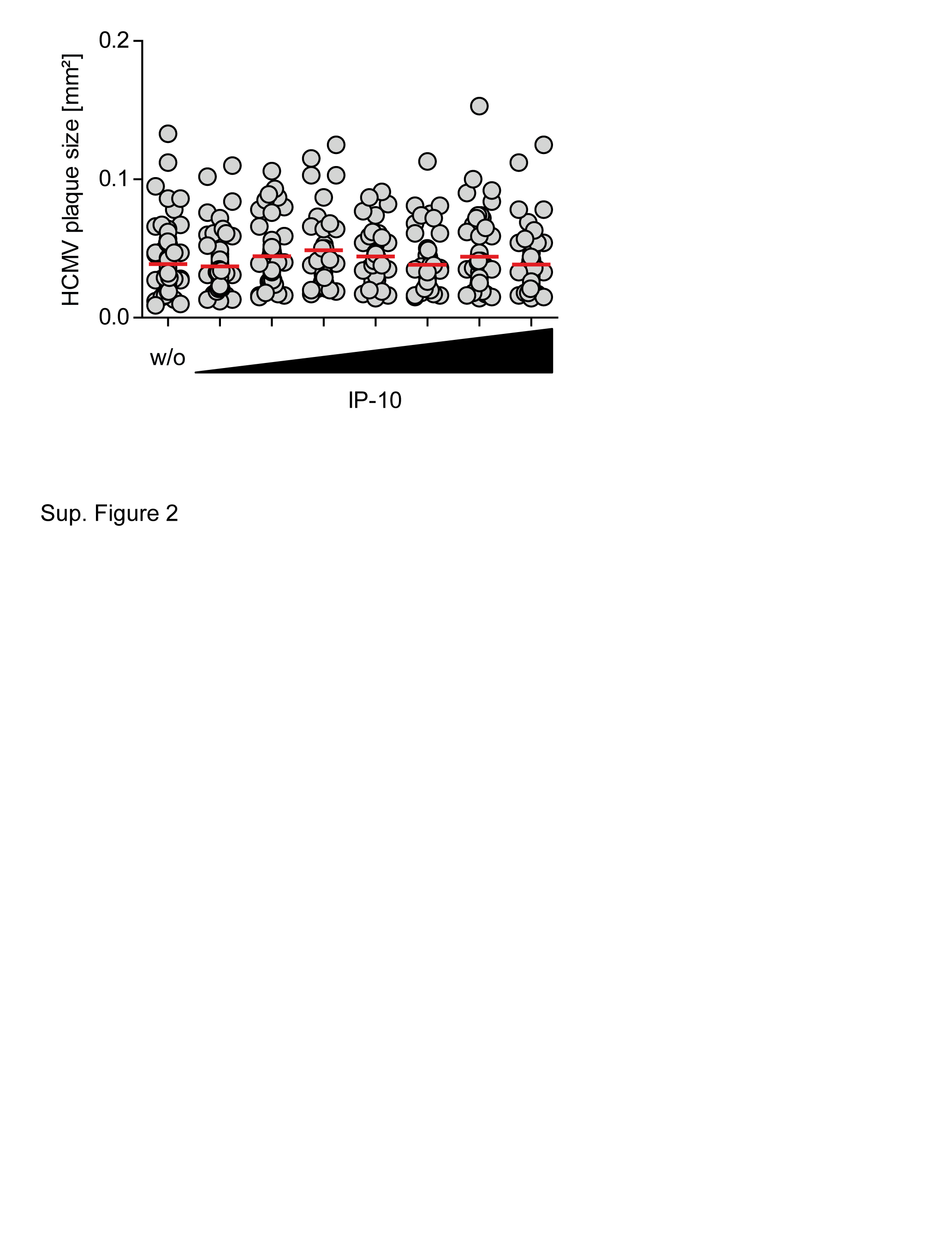


**Sup. Figure 2: Treatment with rec. IP-10 does not diminish HCMV spread.**

RPE cells were infected with HCMV-GFP at MOI 0.1 and treated with log 3 dilutions ranging from 0.1 to 100 ng/ml of rec. IP-10. HCMV plaque formation was analyzed 8 dpi by fluorescence microscopy and the size of individual plaques was determined. Mean size of 35 – 90 plaques from 3 independent experiments.
